# The Ductus Dilemma: To Close or Not to Close in the Fight Against Endocarditis/Endarteritis

**DOI:** 10.3390/children13030340

**Published:** 2026-02-27

**Authors:** Tessa A. E. Soede, Gabriëlle G. van Iperen, Johannes M. P. J. Breur

**Affiliations:** Center for Congenital Heart Disease Utrecht, Wilhelmina Children’s Hospital, University Medical Center Utrecht, 3584 EA Utrecht, The Netherlands; t.a.e.soede-2@umcutrecht.nl (T.A.E.S.);

**Keywords:** patent ductus arteriosus, infective endocarditis, infective endarteritis, congenital heart disease, percutaneous PDA closure, pediatric cardiology

## Abstract

**Highlights:**

**What are the main findings?**

- Infective endocarditis is rare in patients with an audible and non-audible persistent arterial duct.
- The benefits of ductal closure do not outweigh the drawbacks in cases involving hemodynamically insignificant ducts.

**What is the implication of the main finding?**

- Hemodynamically insignificant ducts should not be closed.

**Abstract:**

Background: Patent ductus arteriosus (PDA) is a common congenital heart defect. While closure of hemodynamically significant PDAs is well established, closure of small, hemodynamically insignificant PDAs for prevention of infective endocarditis or endarteritis (IEE) remains controversial and is supported only by low-level evidence. Methods: A systematic PubMed search was performed in May 2025 to identify published case reports of PDA-associated IEE. Data on PDA characteristics, audibility, vegetation location, and causative pathogens were extracted. In addition, the annual national number of percutaneous PDA closures in The Netherlands was determined using data from the Dutch Society of Pediatrics. Finally, the literature was searched for the incidence of complications of percutaneous ductal closure. Results: Seventy-two PDA-IEE cases were identified, of which fifty-five reported PDA audibility. Most cases involved audible PDAs with high-velocity turbulent flow and vegetations at sites exposed to shear stress, particularly the main pulmonary artery. Silent PDAs demonstrated similar vegetation locations and flow characteristics, suggesting that they had comparable pathophysiology. National data showed an average of 76.6 percutaneous PDA closures annually, of which 61% were hemodynamically insignificant. Adverse events during percutaneous ductal closure occur in 23.3% of procedures and clinically significant complications are reported in 10.1%. Both audible and silent PDAs appear capable of promoting IEE through similar hemodynamic mechanisms. Conclusions: Given the low incidence of PDA IEE relative to procedural risks, the high number needed to treat and the associated costs, routine closure of non-hemodynamically significant PDAs solely for IEE prevention is not clearly justified, and no distinction should be made between audible and silent PDAs. The current guidelines warrant critical reassessment.

## 1. Introduction

The ductus arteriosus is a normal fetal blood vessel that connects the descending aorta to the main pulmonary artery. After birth, this shunt becomes redundant, and in most healthy newborns, the ductus closes spontaneously within 12 to 24 h of life [1]. When the ductus fails to close completely, it results in a condition known as patent ductus arteriosus (PDA), a common congenital heart defect. In the general population, the incidence of PDA is estimated at roughly 1 in 2000 live births [2]. The clinical relevance of a PDA depends on its size and shunt magnitude: large PDAs may cause left-sided volume overload and heart failure, whereas small PDAs are often asymptomatic [3]. Very small PDAs are often referred to as “(clinically) silent” as they produce little to no murmur, cause no symptoms, and present with no other clinical signs. Such silent PDAs may go unnoticed until they are incidentally detected through screening or echocardiography performed for unrelated reasons [4].

According to the American Heart Association (AHA) guidelines, transcatheter closure is strongly recommended for moderate-sized or large PDAs with a left-to-right shunt causing heart failure, pulmonary over-circulation, or left heart enlargement [5]. This recommendation is supported by Level A evidence, based on data derived from multiple randomized clinical trials or meta-analyses (class I recommendation). For small PDAs with a left-to-right shunt and normal heart size, closure is considered reasonable when the ductus is audible on auscultation (class IIa recommendation). According to the AHA guidelines, transcatheter PDA occlusion may be considered for PDAs with a small left-to-right shunt, normal heart size, and an inaudible murmur (class IIb recommendation). The latter two recommendations concern hemodynamically insignificant PDAs and are based on Level C evidence, reflecting expert consensus, case studies, or standard of care. As a result, many hemodynamically insignificant but audible PDAs discovered incidentally during screening or echocardiographic evaluation are closed during percutaneous intervention, whereas silent PDAs are often not closed. According to the AHA guidelines, in addition to closure for hemodynamic reasons, an indication for PDA closure is the need to prevent PDA-associated infective endocarditis or endarteritis (PDA-IEE).

IEE is an infection of the endothelium and develops through a sequence of endothelial injury, bacteremia, bacterial adhesion, and in situ persistence. Healthy endothelium is normally resistant to bacterial colonization; however, abnormal flow patterns associated with a PDA generate non-physiological shear stress [6]. Common causative organisms, such as Staphylococcus aureus, possess specific adhesins that allow them to adhere to the endothelium [7]. Studies have shown that shear stress outside the normal physiological range induces upregulation of lectin-type oxidized low-density lipoprotein receptor 1 (LOX-1) expression [8]. This upregulation facilitates binding of bacterial wall teichoic acids (WTAs) to the receptor [9]. Shear stress also increases the release of the von Willebrand factor (VWF) by human vascular endothelium [10], which acts as a binding partner for staphylococcal protein A [11]. Shear stress also induces vascular matrix remodeling, in which fibronectin reorganization plays a key role [12], thereby increasing its availability for interaction with fibronectin-binding proteins of S. aureus [13]. Also, fibronectin from ECM may become available as a binding partner due to endothelial damage caused by shear stress. Collagen can also become available as a binding partner for bacterial collagen-binding adhesins in this way. Under high-shear-stress conditions, S. aureus adheres even more strongly to collagen [14]. Together, these mechanisms suggest that the altered hemodynamic environment of a PDA may facilitate endothelial colonization and subsequent IEE.

The guideline recommendations highlight important gaps in the current evidence base. The benefit of closing hemodynamically insignificant but audible PDAs for the prevention of IEE remains uncertain, and the rationale for differentiating between audible and silent PDAs is not well established. Given that percutaneous PDA closure is an invasive procedure, while IEE is a rare but potentially fatal complication, a clear understanding of the balance between risks and benefits is essential. This review therefore examines the available evidence to determine to what extent percutaneous closure of a hemodynamically insignificant PDAs in pediatric patients reduces the risk of developing IEE, in order to determine whether this represents optimal care.

## 2. Materials and Methods

### 2.1. Case Report Research

A comprehensive literature search for previously described PDA-IEE cases was conducted using the PubMed database in May 2025. The following search terms were used: “patent ductus arteriosus AND endarteritis” and “patent ductus arteriosus AND endocarditis”. A filter for case reports was applied. In cases where two relevant patient reports were described within a single article, both were included. Articles were excluded if full-text access was not available online, even via the Utrecht University library. Additional exclusion criteria were: reports on unrelated topics, cases of IEE occurring after PDA closure, or studies involving animal subjects. No publication date restrictions were imposed.

The following information was extracted from the articles: author, publication year, patient age, PDA size, PDA flow, whether the PDA was silent or not, pathogen, and location of the vegetation. The assessment of silent PDAs was made based on the following criteria:

- If the article explicitly stated “silent PDA” → silent.
- If the article stated “no past history of a heart murmur” → silent.
- If the article stated “a new murmur” → silent.
- If the article stated “heart murmur was previously known” → not silent.
- If the article stated “heart murmur was heard, consistent with a PDA” → not silent.
- If the article stated “continuous murmur was best heard in the second left intercostal space” → not silent [15].

### 2.2. National Case Volume

To estimate the number of annual percutaneous PDA closures in The Netherlands, data from the Dutch society of Pediatrics were used. The National Working Group for Cardiac Catheterization in Congenital Heart Disease has prepared documents containing information on cardiac catheterizations performed in the Netherlands in children and adults with a congenital heart defect [16]. From these documents, the number of PDA closures was extracted for each center. For each year, all closures were summed, and then an annual average was calculated.

To distinguish between hemodynamically significant and insignificant PDA closures, for the same years used to calculate the annual number of closures, the catheterization reports from the Wilhelmina Children’s Hospital were reviewed to determine whether the PDA was hemodynamically significant or insignificant. Based on this, a percentage was calculated to represent the distribution between the two.

### 2.3. Incidence of Complications of Percutaneous Ductal Closure

To determine the incidence of complications of percutaneous ductus closure, a search of the literature was conducted.

## 3. Results

### 3.1. Case Report Research

**Figure 1:**
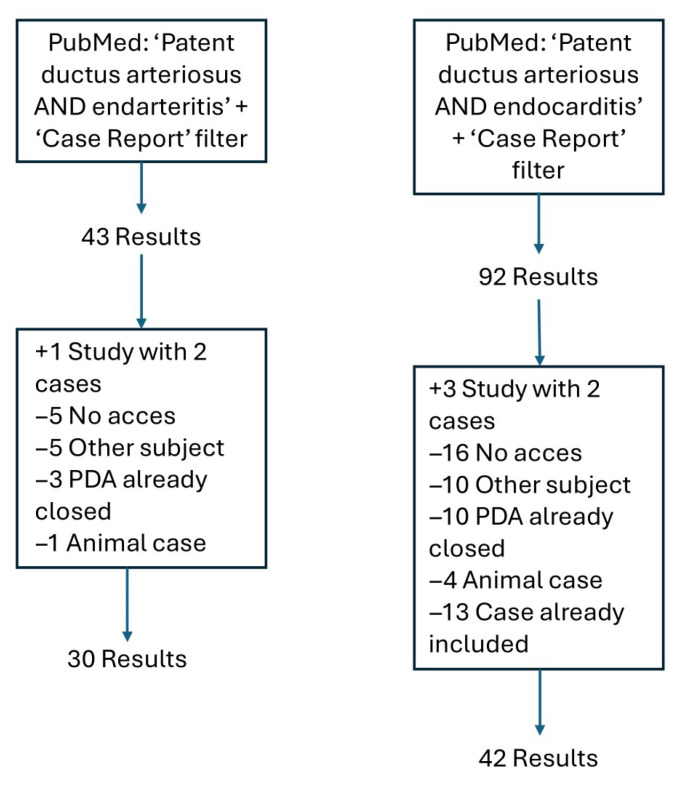
Study flow diagram detailing the process undertaken during the literature search for this review. The search entered into PubMed yielded 122 articles. Of these, 4 articles described two relevant patient reports, 21 were excluded because full-text access was not available, 15 articles concerned other topics, 13 articles reported IEE in a PDA that had already been closed, and 5 were excluded because they described an animal case.

In total, 72 cases of PDA-IEE have been described, of which 55 cases reported whether the PDA was clinically silent or not (Figure 1) (Appendix A). The publication years ranged from 1980 to 2025.

Of these 55 cases, 80% (n = 44) involved an audible PDA. Fifty-one of these cases provided information regarding the size and/or flow characteristics of the PDA. Most of these PDAs were described as “large” or measured between 5 and 10 mm in diameter, but there were also cases described as “small” or measuring 3 mm. In the majority of the audible cases, the PDA flow was characterized as “high-velocity turbulent flow”. In 40 of the 44 cases, the location of the vegetation was also reported. The most common sites were the main pulmonary artery (n = 28), pulmonary valve (n = 9), aortic valve or aorta (n = 5), left pulmonary artery (n = 5), the PDA itself (n = 4), mitral valve (n = 3), and other locations (n = 2). The total number of vegetation growths exceeded 40, as some cases involved multiple vegetations in different locations and each one was categorized individually.

The remaining 20% (n = 11) involved clinically silent PDAs. Among these cases, ten provided information regarding the size and/or flow characteristics of the PDA. Variable PDA size was reported, ranging from 1 to 6 mm. Flow characteristics were described in most cases as a tiny jet flow, but in others as high-velocity turbulent flow. Vegetation was predominantly located in the main pulmonary artery (n = 6), within the ductus itself (n = 4), on the aortic valve (n = 3), or on the pulmonary valve (n = 3).

Of the 72 documented cases, the causative pathogen was identified in 54 cases. The most frequently reported pathogens were S. aureus, Streptococcus viridans and Streptococcus sanguis.

During the exclusion process, 13 cases were excluded because the PDA was already closed at the time the endocarditis occurred. Full-text access was available for 11 of these cases. The articles provided the following information regarding the closure of the PDA: the PDA had closed spontaneously (n = 1) [17]; the PDA had been surgically or percutaneously closed with no residual shunt (n = 7) [18,19,20,21,22,23,24]; or the PDA had been surgically or percutaneously closed but a residual shunt remained (n = 3) [25,26,27]. In the cases with a shunt, the vegetation was located in the main pulmonary artery (n = 3) or the left pulmonary artery (n = 1). Among the seven cases in which the PDA had been completely closed surgically, endocarditis or endarteritis developed in four cases within 5 weeks after surgery. In the remaining cases, the infection occurred 6 to 11 years after the operation. In this latter group, vegetations were described as a narrowing of the aorta, left ventricle, and aortic valve.

### 3.2. National Case Volume

Data on percutaneous interventions for congenital heart disease were evaluated for the years 2017 through 2021. In 2017 there were 73 closures; in 2018, there were 71; in 2019, there were 75; in 2020, there were 75; and in 2021, there were 89. The average number of procedures performed in the Netherlands during these years was 76.6 per year. This included procedures performed on both children and adults.

The catheterization reports from Wilhelmina Children’s Hospital show that 39% of PDA closures involved hemodynamically significant PDAs, while the remaining 61% involved hemodynamically insignificant PDAs.

### 3.3. Incidence of Complications of Percutaneous Ductal Closure

A meta-analysis of the literature demonstrated a technical success rate of 92.2% for percutaneous PDA closure [28]. Procedures were primarily aborted for the following reasons: adverse events (AEs) such as bleeding or arrhythmia, device malposition within the aorta, device malposition within the left pulmonary artery, and technical failure. AEs were reported in 23.3% of cases, with 10.1% classified as clinically significant. Device- or coil-related complications were identified as the most common type of AE in a meta-analysis, and typically involved embolization or malposition. Embolizations were primarily observed within the pulmonary arteries and the aorta. In addition, a substantial number of access-related AEs were reported. These included hematoma or transient pulse loss that did not require therapy; pulse loss or thrombosis requiring treatment; and blood transfusion due to vascular compromise.

## 4. Discussion

In audible PDA cases, the ductus is typically large and associated with high-velocity turbulent flow, which generates significant shear stress. This stress likely contributes to the formation of vegetations within the PDA itself, as well as in the wall of the main pulmonary artery, particularly near the origin of the left PA where the turbulent jet usually impacts. The flow through the ductus into the pulmonary artery can also increase the pressure and mechanical stress on the pulmonary valve, providing a plausible explanation for vegetations observed at that site. Furthermore, as blood shunts into the pulmonary circulation and returns to the left heart, volume overload may develop, potentially affecting the mitral and aortic valves, which could account for the vegetations found in those regions. Together, these factors provide a plausible mechanistic explanation for the locations of vegetations observed in audible PDAs, supporting the notion that the ductus acts as a causative factor in IEE.

In cases involving silent PDAs, there was considerable variation in both the size of and the flow through the ductus. The vegetations found in cases with silent PDAs correspond to the locations observed in cases with audible PDAs. Based on PDA size, flow characteristics, and vegetation location, there does not appear to be a clear difference between audible and silent PDAs. This suggests that the underlying pathophysiological mechanisms are comparable.

The classification of “silent” PDAs is inherently ambiguous. A shunt may be too small or, paradoxically, too large to produce detectable turbulence. A small diameter can also lead to a high flow velocity and, thus, increased shear stress. Furthermore, the detection of murmurs relies on clinical auscultation, which is subjective and can vary between clinicians. Turbulent jets may also be misclassified as silent if the flow direction prevents them from being heard with a stethoscope. This challenging classification process, combined with the similarities in vegetation location between silent and audible PDAs, and the variation in size and flow observed in silent PDAs in the case report study, makes it unjustifiable to treat silent and audible PDAs as fundamentally separate categories in clinical guidelines. Although some characteristics may differ between the groups, there is no evidence to support managing these two groups differently. Both should be managed in the same way.

In the case of a residual shunt PDA, the residual flow may have caused turbulent flow and shear stress, potentially leading to IEE. Among the cases in which the PDA had been completely closed surgically, three IEEs developed shortly after surgery. This may indicate that PDA closure itself also carries a risk of IEE. In the remaining cases, the infection occurred years after the operation. Research has shown that endothelial healing after mechanical injury typically occurs within seven days [29]. Furthermore, the locations where vegetations were observed in these cases do not correspond to the typical sites associated with PDA-IEE. These observations suggest that the PDA was unlikely to have been the causative factor in these cases, and that PDA-IEEs reported in the literature may be overestimated.

Although a PDA appears to increase the risk of IEE, any potential benefit of closure must be weighed against the incidence of PDA IEE, the additional healthcare costs, and the adverse events associated with the closure procedure. A Canadian study reported a lesion-specific incidence of IE in patients with a PDA of 0.24 per 1000 patient-years, equivalent to approximately 1 case per 4166 patient-years [30]. As Canada is a developed country, the general incidence of IE is estimated at 22.5 (between 15 and 30) cases per million individuals per year, or approximately 1 case per 44,444 individuals annually (3). Therefore, the incidence of IE in patients with a PDA appears to be at least ten times higher than in the general population. The next step is to apply this incidence to the Dutch population. The Netherlands has approximately 18,073,424 inhabitants [31]. Based on a PDA incidence of 1 in 2000 live births, around 9037 individuals in the population would have a PDA (2). Given a PDA-IE incidence of 1 in 4166 individuals per year, an estimated 2.2 individuals per year in the Netherlands would develop PDA-IE.

Approximately 76.6 percutaneous PDA closures are performed annually in the Netherlands. Based on data from Wilhelmina Children’s Hospital, 61% of these are hemodynamically insignificant, corresponding to 46.7 percutaneous PDA closures. Therefore, the number needed to treat (NNT) per year to prevent one PDA IE is 21.2. The cost per percutaneous PDA closure ranges between €8630 and €14,020, resulting in an annual total cost of €182,956 to €297,224 [32]. Additionally, AEs occur in 23.3% of cases (approximately five Dutch patients annually), with 10.1% (approximately two Dutch patients annually) experiencing a clinically significant AE. Taken together, these figures highlight that percutaneous PDA closure carries substantial risks and costs, with many patients potentially experiencing harm without clear benefit.

The guideline, developed by cardiologists, does not cite evidence demonstrating that PDA closure reduces the risk of IEE. Instead, the recommendations are based on expert consensus, case reports, or standard-of-care practices, with closure of an audible PDA classified as Class IIa and closure of a silent PDA as Class IIb. These considerations raise concerns regarding the objectivity and the supporting evidence for the recommendations. While mechanistic reasoning and case-based observations suggest that a PDA may increase the risk of IEE, the literature shows that the benefits of closure do not outweigh the harms. The incidence of PDA IE is low, the NNT is high, AEs are frequent, and the costs are substantial. Therefore, such procedures should no longer be performed, and PDA closure for IEE prevention should be reclassified as a class III recommendation in the next AHA guideline.

One limitation of this study is that, for all years analyzed, the annual number of percutaneous PDA closures at the Center for Congenital Heart Disease Amsterdam–Leiden (CAHAL) includes ductus closures reported together with Major Aortopulmonary Collateral Arteries (MAPCAs) closures. As a result, the exact number of PDA closures cannot be determined. The same applies to UMC Utrecht/WKZ/Nieuwegein for the years 2020 and 2021. Following on from the first year in which MAPCA closures were included at the latter institution, we did not observe an increase in the total number of reported closures. Furthermore, MAPCA closure is performed only sporadically; therefore, this is likely to result in only a minor overestimation of the number of PDA closures.

## 5. Conclusions

Both pathophysiological mechanisms and case reports indicate that a PDA creates an environment conducive to IEE development, with audible and silent PDAs posing comparable risk. Closure of the PDA eliminates this environment and could therefore reduce the risk. When closure is complete and there is no residual shunt, IEE has been reported at atypical sites and times, suggesting a loss of causal association. However, considering the low incidence of PDA IEE, the high number of AEs, the high NNT, and the associated costs, current clinical guidelines warrant critical reassessment. Based on available evidence, routine closure of non-hemodynamically significant PDAs solely for IEE prevention is not clearly justified, and no distinction between audible and silent PDAs should influence management. In the next AHA guideline, both audible and silent PDA closure should be classified as a class III recommendations.

## Data Availability

No new data were created or analyzed in this study.

## Abbreviations

The following abbreviations are used in this manuscript:

AE	Adverse Event.
AHA	American Heart Association.
CAHAL	Center for Congenital Heart Disease Amsterdam-Leiden.
IEE	Infective Endocarditis/Endarteritis.
LOX-1	Low-Density Lipoprotein Receptor 1.
MAPCA	Major Aortopulmonary Collateral Arteries.
PDA	Patent Ductus Arteriosus.
PDA-IEE	Patent Ductus Arteriosus-associated Infective Endocarditis/Endarteritis.
VWF	Von Willebrand Factor.
WTAs	Wall Teichoic Acids.

## Appendix A

**Table A1:** Literature overview of case reports with PDA-IEE, sorted by year. The relevant data from the articles has been copied directly. x = not extractable from the article; y = yes; n = no; w = weeks; m = months; y = years.

Author and Reference	Year	Age	PDA Size	PDA Flow	Silent	Pathogen	Location Vegetation
Nakamura [33]	1980	40 y	x	Pulmonary to systemic flow ratio of 2.9:1	n	*Streptococcus viridans*	x
Bianchi [34]	1982	9 y	x	x	n	*Streptococcus viridans*	Pulmonary valve leaflet
Vargas-Barron [35]	1985	9 y	15 mm long and 6 mm wide	Turbulent flow	n	x	Lateral wall of pulmonary artery
Thomas [36]	1987	74 y	x	x	n	*Streptococcus bovis*	x
Stejskal [37]	1992	7 y	Small	x	n	*Streptococcus viridans*	Pulmonary artery wall opposite the mouth of the duct
Balzer [38]	1993	19 y	x	Tiny jet of retrograde flow	y	*Staphylocccus aureus*	Left pulmonary artery entrance of the PDA
Tsukui [39]	1998	x	x	x	x	*Steptococcus*	x
Sánchez [40]	1998	10 y	x	x	x	*Coxiella burnetii*	Aortic valve
Berdagué [41]	1998	15 y	x	x	x	*Bartonella*	Pulmonary valve
Parthenakis [42]	2000	18 y	x	Tiny jet of retrograde flow in the main pulmonary area	y	*Streptococcus sanguis* II	Left pulmonary artery entrance of the PDA (left)
Yanýk [43]	2000	14 y	x	x	n	*Streptococcus viridans*	Just proximal to the bifurcation of the pulmonary artery + opening side of the patent ductus arteriosus
Kouris [44]	2003	43 y	x	High-velocity retrograde turbulent flow (4 m/s) emerging from the descending aorta towards the pulmonary artery at the level of its bifurcation	n	*Streptococcus sanguis* II	Pulmonary artery wall + pulmonary end of the PDA
Kadakia [45]	2004	25 y	x	A high-velocity jet	n	*Streptococcus mutans*	Main pulmonary artery just superior to the pulmonary bifurcation
Bilge [46]	2004	11 y	x	Continuous flow from the aorta to the pulmonary artery across ductus with a peak velocity of 4 m/s.	n	*Streptococcus viridans*	Wall of pulmonary artery, just proximal to the origin of the left pulmonary artery
Shuhaiber [47]	2004	34 y	Large (10 mm diameter)	Left-to-right shunt	x	*Staphylococcus epidermidis*	Aortic valve + pulmonary valve
Ozkokeli [48]	2004	27 y	5 mm in diameter	Turbulent pattern	y	*Alpha-hemolytic streptococcus*	Noncoronary cusp of the aortic valve + pulmonary valve + wall of pulmonary artery
Cerruto [49]	2005	50 y	x	x	y	*Corynebacterium pseudodiphteriticum*	Noncoronary aortic valve cusp
Cruz-González [50]	2006	56 y	x	x	n	*Streptococcus viridans*	Wall of the pulmonary artery
Malnick [51]	2006	31 y		x	n	*Streptococcus anginosus*	Left pulmonary artery
Onji [52]	2007	49 y	2 mm in length	High-velocity, turbulent retrograde flow emerging from the descending aorta towards the pulmonary artery at the level of its bifurcation	y	*Streptococcus mitis*	Pulmonary artery
Celebi [53]	2007	2 m	x	Very tiny retrograde turbulent flow into the left side of the main pulmonary artery	y	x	Left side of the wall of the main pulmonary artery
Aggarwal [54]	2007	25 y	x	x	n	x	Leaflets of aortic and pulmonary valves + lateral wall of the pulmonary trunk
Grover [55]	2008	1 w	1 mm	Hemodynamic insignificance	y	*Staphylocccus*	Pulmonary insertion of the closed ductus arteriosus
Grover [55]	2008	1 w	Small	x	n	x	x
Kiani [56]	2008	5 y	Large	x	x	x	Main pulmonary artery at the site of patent ductus arteriosus jet flow
Satoh [57]	2008	92 y	5 mm diameter	Spectral flow pattern from the descending aorta to the main pulmonary artery via PDA and continuous-wave Doppler flow in the ducts	n	Gram-positive cocci	Mitral anterior leaflet + aortic valve
Bathoorn [58]	2009	62 y	x	Abnormal flow from the aorta to the pulmonary artery through a PDA	n	x	Pulmonary trunk downstream of the PDA
Cagli [59]	2010	27 y	x	High-velocity, turbulent flow emerging from the descending aorta toward the pulmonary artery (PA) at the level of its bifurcation,	n	Gram-positive cocci	Aortic valve + mitral valve + in the path of blood passing through the PDA to the PA
Song [60]	2010	16 y	18 mm long and 8 mm wide	Peak velocity was 4.5 m/s and diastolic flow was 3 m/s	n	None (non-infective endocarditis)	Wall of the pulmonary artery on the downstream side of the PDA + right coronary cusp of the aortic valve
Kareem [61]	2010	9 y	x	x	n	x	Main pulmonary artery adjacent to the patent ductus arteriosus
Navaratnarajah [62]	2011	34 y	Large, maximal diameter 16 mm and length 25 mm	High-velocity retrograde flow from the descending aorta towards the main pulmonary artery bifurcation in the parasternal short-axis view	n	*Streptococcus viridians*	Main pulmonary Artery
Ferreira [63]	2011	4 m	Moderate	x	y	*Klebsiella pneumoniae*	Wall of ductus arteriosus
Matsukuma [64]	2011	35 y	x	x	x	*Staphylococcus aureus*	In the dilated pulmonary artery + pulmonary arterial wall near the orifice of the duct
Gokaslan [65]	2011	9 y	Large	x	n	x	Anterior wall of the main pulmonary artery + ventricular surface of the septal pulmonary leaflet
Souaga [66]	2012	7 y	3 mm diameter; restrictive	x	n	Beta-hemolytic streptococcus	Pulmonary valves + origin of left pulmonary artery
Souaga [66]	2012	5 y	6 mm diameter	Restrictive flow	n	x	Pulmonary valves + trunk of pulmonary artery
Srivastava [67]	2012	5 y	2.3 mm at the narrowest portion	Shunting from left to right with a gradient of 76/12 mmHg across	x	x	x
Sugimura [68]	2013	63 y	x	Continuous jet from the aortic arch to the pulmonary artery	y	*Pseudomonas aeruginosa*	Pulmonary arterial wall near the orifice of the duct
Kim [69]	2014	50 y	6 mm	The amount of shunt flow was too small to measure	y	x	Left coronary cusps + in the pulmonary artery + at the site opposite to the ductal opening
Sabzi [70]	2015	28 y	Large diameter (10 mm)	Small shunt	n	*Streptococcus pneumoniae*	Intra-luminal on the main pulmonary artery side of the PDA
Mishra [71]	2016	7 y	2 mm at the pulmonary arterial end	High-velocity turbulent flow	n	x	Exit of LMCA into right atrium
Shi [72]	2016	10 y	5 mm shunting duct	x	n	*Staphylocccus aureus*	Left side of pulmonary artery trunk
Malviya [73]	2016	7 y	Large aneurysmal	Left-to-right shunt	x	x	On pulmonary end of the duct + both branch pulmonary arteries + right atrial surface of interatrial septum
Miraclin [74]	2017	54 y	x	x	n	*Abiotrophia defectiva*	Main pulmonary artery at the site of PDA
Siddiqa [75]	2018	40 y	x	Continuous flow of PDA. Pressure gradients showed end diastolic velocity of PDA 2.6 m/s.	n	x	Main pulmonary artery at the side of patent ductus arteriosus
Sattwika [76]	2018	26 y	x	Bidirectional with predominant left-to-right shunt perimembranous VSD and PDA	n	*Streptococcus viridans*, *Streptococcus procinus*, *Streptococcus mitis*/*oralis*	Pulmonary valve
Sattwika [76]	2018	32 y	x	x	n	x	Pulmonary valve + wall of pulmonary artery + VSD
Lee [77]	2019	42 y	x	x	x	x	Junction of the left main pulmonary artery and patent ductus arteriosus
Wang [78]	2019	35 y	x	Bidirectional cardiac shunting with dominant left-to-right shunting.	x	Gram-positive bacilli	Left ventricular outflow tract
Ramiro [79]	2019	19 y	Moderate, PSAX diameter 9 mm, 10 mm (with color); suprasternal 8.1 mm, 1 mm (with color).	Qp:Qs of 1.18	x	*Streptococcus viridans*, psedomonas putida	Pulmonary artery + pulmonic valve
Chen [80]	2019	49 y	x	x	x	*Enterococcus faecalis*	Aortic valve
Micovic [81]	2020	56 y	4–5 mm	x	x	*Staphylococcus aureus*	x
Wang [82]	2020	30 y	x	Left-to-right shunt	n	Streptococcus Gram-positive group G	Beginning of the left pulmonary artery + mitral valve
Batta [83]	2021	40 y	6 mm	Left-to-right shunt across PDA with a peak gradient of 54 mm Hg and an end diastolic gradient of 28 mm Hg.	n	*Streptococcus mutans*	Just proximal to the bifurcation of the pulmonary artery, where the PDA jet struck the pulmonary artery
Mahmood [84]	2021	3 y	x	x	x	*Straphyloccus aureus*	Main pulmonary artery + normal coronaries
Gwak [85]	2022	52 y	8.5 mm	Peak velocity 4.5 m/s	n	*Streptococcus sanguinis*	Main pulmonary artery
Bouhdadi [86]	2022	23 y	10 mm (Large)	Left-to-right shunt	n	*Streptococcus viridans*	Fixed structure on wall of pulmonary artery + wall of the descending aorta in the supra-sternal view
Mahajan [87]	2022	10 y	x	x	n	*Pseudomonas aeruginosa*	Pulmonary valve + main pulmonary artery’s wall
Miyagami [88]	2022	33 y	x	x	n	*Streptococcus oralis*	Patent ductus arteriosus area
Wu [89]	2023	10 y	Pulmonary artery end 5.8 × 4.8 mm	Left-to-right shunt flowing at 4.2 m/s	n	*Micrococcus luteus*	Left pulmonary artery
Kanai [90]	2023	33 y	x	x	n	*Streptococcus oralis*	PDE edge on the side of the pulmonary artery
Hidaka [91]	2023	24 y	x	x	x	x	Pulmonary artery + pulmonic valve
Canning [92]	2024	9 y	Moderate-sized	Left-to-right shunt	y	*Streptococcus sanguinis*	Main pulmonary artery, where the PDA jet was directed to
Shakya [93]	2024	9 y	3.0 mm pulmonary end	Hemodynamically significant	n	x	Main pulmonary artery
Mayer [94]	2024	1 w		Hemodynamic significance	x	*Enterococcus faecalis*	Inside the ductus
Mekonnen [95]	2024	10 y	Small	Continuous left-to-right shunt and peak pressure gradient of 50 mm Hg	n	*Klebsiella pneumoniae*	At the pulmonic end
Borcan [96]	2024	37 y	x	x	x	*Aggregatibcter actinomycetemcomitans*, *Klebsiella pneumoniae*	Lateral wall of the pulmonary artery trunk + pulmonary valve
Dam [97]	2024	38 y	5.8 mm in left pulmonary input and 7.6 mm in the aortic side	Left-to-right shunt	n	*Staphylocccus arueus*	Left main pulmonary artery
Barrerra-Colín [98]	2024	8 y	Pulmonary opening of 3 mm	x	n	Nutritionally variant streptococci	In truncus pulmonalis
Hu [99]	2025	45 y	x	x	n	*Streptococcus* spp.	Aortic valve
Zhang [100]	2025	7 y	Narrowest width of 5 mm	x	n	*Staphyloccocus aureus*	x
Munawar [101]	2025	18 y	Small	x	n	*Staphylococcus aureus*	Main pulmonary artery
